# Genetic diversity and virulence potential of clinical and environmental *Aeromonas* spp. isolates from a diarrhea outbreak

**DOI:** 10.1186/s12866-017-1089-0

**Published:** 2017-08-18

**Authors:** Lívia Christina Alves da Silva, Tereza Cristina Leal-Balbino, Beatriz Souza Toscano de Melo, Carina Lucena Mendes-Marques, Antonio Mauro Rezende, Alzira Maria Paiva de Almeida, Nilma Cintra Leal

**Affiliations:** Instituto Aggeu Magalhães (IAM/Fiocruz PE), Avenida Professor Moraes Rego, s/n. Cidade Universitária, Recife, Pernambuco 50740-465 Brazil

**Keywords:** *Aeromonas*, Taxonomy, Virulence, Diarrhea, Outbreaks

## Abstract

**Background:**

*Aeromonas* spp. are gram-negative bacteria that can cause a variety of infections in both humans and animals and play a controversial role in diarrhea outbreaks. Our aim was to identify clinical and environmental *Aeromonas* isolates associated with a cholera outbreak in a northeast county of Brazil at the species level. We also aimed to determine the genetic structure of the bacterial population and the virulence potential of the *Aeromonas* isolates.

**Methods and results:**

Analysis based on concatenated sequences of the 16S rRNA and *gyrB* genes suggested the classification of the 119 isolates studied into the following species: *A. caviae* (66.9%), *A. veronii* (15.3%), *A. aquariorum* (9.3%), *A. trota* (3.4%), *A. hydrophila* (3.4%) and *A. jandaei* (1.7%). One isolate did not fit any *Aeromonas* species assessed, which might indicate a new species. The haplotype network based on 16S rRNA gene sequences identified 59 groups among the 119 isolates and 26 reference strains, and it clustered almost all *A. caviae* isolates into the same group. The analysis of the frequency patterns of seven virulence-associated genes (*alt*, *ast, hlyA*, *aerA*, *exu*, *lip*, *flaA*/*B*) revealed 29 virulence patterns composed of one to seven genes. All the isolates harbored at least one gene, and three of them harbored all seven virulence genes.

**Conclusion:**

The results emphasize the need to improve local water supply and maintain close monitoring of possible bacterial contamination in the drinking water.

## Background

The genus *Aeromonas* comprises many species of gram-negative bacteria, and it harbors common inhabitants of aquatic environments with some species involved in human and animal infections. In humans, gastroenteritis is the most frequent clinical manifestation of an *Aeromonas* infection. *Aeromonas*-associated diarrhea occurs worldwide in both industrialized and developing countries, affecting all age groups and both healthy and immunocompromised individuals. However, a role for *Aeromonas* in diarrhea outbreaks remains controversial [1, 2].

The *Aeromonas* taxonomy is complex and ever changing due to the introduction of new identification criteria leading to the description of new species and reclassification of known ones [3]. Although analyses based on 16S rRNA gene sequencing are universally accepted to establish phylogenetic relationships for all bacterial genera, the high identity among the 16S rRNA nucleotide sequences of the *Aeromonas* species can impair the taxonomic classification of the *Aeromonas* genus [4, 5]. Therefore, housekeeping genes such as *gyrB* have been used for taxonomic inference of this group [6].

The pathogenesis of *Aeromonas* spp. is also unclear. Many virulence factors involved in different steps of infection mechanisms have been described in different *Aeromonas* species. These virulence factors include (1) fimbriae, flagella, and a capsule that allow attachment to the host surface; (2) toxins and enzymes such as proteases, elastases, lipases, and hemolysins that cause cell and tissue damage; (3) secretion systems that enable evasion of the host immune response; (4) iron-binding proteins called siderophores that scavenge iron from the host; (5) a capsule, S-layer, lipopolysaccharide, and porins that compromise host defenses; (6) biofilm formation that allows adherence to cell surfaces; and (7) quorum-sensing systems that can modulate bacterial virulence gene expression [1–3].

Hofer et al. [7] reported high prevalence of *Aeromonas* spp. as the sole pathogen in diarrheic patient feces, whereas *Vibrio cholerae* O1 prevalence was lower during an alleged cholera outbreak. The infections were attributed to the precariousness of the drinking water supply lacking microbiological control in the region, where a local river both receives untreated sewage and serves as a drinking water source. Despite the higher prevalence of *Aeromonas* isolates in patient feces, *V. cholerae* O1 was entailed as the etiological agent of the outbreak [8]. This issue invited further characterization of those *Aeromonas* isolates. Therefore, this study aimed to identify the clinical and environmental *Aeromonas* outbreak isolates at the species level using a combination of 16S rRNA and *gyrB* gene analysis to elucidate the genetic structure and virulence potential of the *Aeromonas* population.

## Methods

### Bacterial strains

This study involved 119 *Aeromonas* spp. isolates (103 from diarrheal patient feces and 16 from the aquatic environment) obtained from March to June 2004 during a diarrhea outbreak that occurred in São Bento do Una, a Brazilian city located in Pernambuco state [7, 8]. Clinical isolates were numbered from 251 to 374 and environmental isolates were numbered from 420 to 707. The cultures were stored at − 80 °C in Brain Heart Infusion (BHI) broth plus 25% glycerol. The reference strains of *Aeromonas hydrophila* subsp. *hydrophila* ATCC 7966^T^, *Aeromonas veronii* bv. *veronii* ATCC 35624^T^ and *Aeromonas caviae* ATCC 15468^T^ were used as controls.

### DNA extraction

Each culture was inoculated into BHI and incubated at 37 °C for 24 h for DNA extraction, following a protocol based on Leal et al. [9]. Briefly, 1 ml of the culture was centrifuged, and the pellet was homogenized in 500 μl of TE (TrisHCl 10 mmol l^−1^, EDTA 1 mmol l^−1^) pH 8.0 and 10 μl of proteinase K (5 mg ml^−1^) and incubated at 60 °C for 20 min. Next, 100 μl of STE (sodium dodecyl sulfate 2.5%, TrisHCl 10 mmol l^−1^, pH 8.0, EDTA 0.25 mol l^−1^) was added, and the suspension was incubated for 15 min at 60 °C, 5 min at room temperature and 5 min on ice, followed by the addition of 130 μl of ammonium acetate (7.5 mol l^−1^). After 15 min on ice, the material was centrifuged, and the supernatant was transferred to a new tube and purified with phenol:chloroform:isoamyl alcohol (25:24:1). DNA in the upper phase was precipitated with an equal volume of isopropanol. The sediment was suspended in 10 μl RNAse solution (10 mg ml^−1^) and stored at −20 °C.

### Identification of the *Aeromonas* spp. isolates at the species level

The DNA from the 119 *Aeromonas* spp. isolates was analyzed by sequencing the 16S rRNA and *gyrB* genes to identify isolates at the species level. Polymerase Chain Reactions (PCR) were executed as previously described [6, 8]. The amplicons (~1503 base pairs (bp) for the 16S rRNA and ~1100 bp for *gyrB*) were purified using ExoSAP-IT PCR Cleanup (Affymetrix, Cleveland, OH, USA) according to the vendor. The purified products were sequenced using an Applied Biosystems 3100 sequencer (Applied Biosystems, Foster City, CA, USA) using the PCR primers described in Table 1. To guarantee sequence accuracy, each nucleotide sequence was determined at least twice. Sequence assembly and editing were performed using the tools called Pregap4 and Gap4, which belong to the Staden package [10].

**Table 1:** Sequences of the primers used for phylogenetic and virulence potential analysis

Gene	Sequence (5′-3′)	Reference
PCR		
16S rRNAF	AGAGTTTGATCATGGCTCAG	Borrell et al. [19]
16S rRNAR	GGTTACCTTGTTACGACTT	Borrell et al. [19]
*gyr*B 3F	TCCGGCGGTCTGCACGGCGT	Yáñez et al. [20]
*gyr*B 14R	TTGTCCGGGTTGTACTCGTC	Yáñez et al. [20]
*hly*AF	GGCCGGTGGCCCGAAGATACGGG	Heuzenroeder et al. [21]
*hly*AR	GGCGGCGCCGGACGAGACGGG	Heuzenroeder et al. [21]
*alt*F	CCATCCCCAGCCTTTACGCCAT	Martínez et al. [22]
*alt*R	TTTCACCGAGGTGACGCCGT	Martínez et al. [22]
*ast*F	ATGCACGCACGTACCGCCAT	Martínez et al. [22]
*astR*	ATCCGGTCGTCGCTCTTGGT	Martínez et al. [22]
Sequencing		
16S BF	AGCAGTGGGGAATATTGCAC	This study
16S BR	GGCAACAAAGGACAGGGGT	This study
16S CF	ACGCAGGCGGTTGGATAAGT	This study
16S CR	AAATCGACATCGTTTACGGCG	This study
16S DF	AACCTTACCTGGCCTTGACA	This study
16S DR	CACACACGCGGCATGGTGCATC	This study
gyrB 9Rs	CCTTGACCGAAATGACCGCC	Yáñez et al. [20]
*gyr*B 7F	GGGGTCTACTGCTTCACCAA	Yáñez et al. [20]
*gyr*B 9R	ACCTTGACGGAGATAACGGC	Yáñez et al. [20]

Sequences of 1454 bp and 920 bp for 16S rRNA and *gyrB* genes, respectively, were used in the final analysis. These partial gene sequences were aligned using the Muscle program [11] independently and as concatenated sequences (2374 bp) with those from reference strains of published *Aeromonas* sequences available from the National Center for Biotechnology Information (NCBI) database at the time this study was conducted. *Salmonella enterica* ATCC 13311 T was included when outgroup rooting was needed for the analysis, as the two genera are closely related. The GenBank accession numbers of the utilized sequences are listed in Table 2.

**Table 2:** Accession numbers of the 16S rRNA and *gyrB* sequences of type strains of *Aeromonas* spp.

Type strains	16S rRNA accession numbers	*gyrB* accession numbers
*A. molluscorum* LMG 22214	AY987772.1	AY987538.1
*A. tecta* CECT 7082	HQ832416.1	HQ442662.1
*A. media* ATCC 33907	X74679.1	AF417627._1
*A. eucrenophila* ATCC 23309	X74675.1	AF417629.1
*A. encheleia* CECT 4342	HQ832414.1	Ay101799.1
*A. popoffii* CECT 5176	HQ832415.1	AY101801.1
*A. bestiarum* LMG 13444	AY987755.1	AY987521.1
*A. piscicola* CECT 7443	HQ832417.1	HQ442690.1
*A. schubertii* ATCC 43700	X60416.2	AF417628._1
*A. simiae* MDC 2374	GQ860944.1	GQ860942.1
*A. jandaei* ATCC 49568	X60413.2	AF242651.3
*A. trota* ATCC 49657	X60415.2	AF417633.1
*A. salmonicida* CECT 894	Ay987751.1	JN711820.1
*A. allosaccharophila* CECT 4199	S39232.2	HQ442733.1
*A. aquariorum* MDC 47	EU085557.2	EU268444.1
*A. diversa* CECT 4254	GQ365710.1	HQ442756.1
*A. rivuli* DSM 22539	FJ976900.1	FJ969434.1
*A. bivalvium* 868E	NR_043885.1	EF465525.1
*A. fluvialis* CECT 7401	FJ230078.2	FJ603455.1
*A. sobria* JCM 2139	AB472942.1	AB473084.1
*A. sanarellii* CECT 7402	FJ230076.1	FJ807277.1
*A. taiwanensis* CECT 7403	FJ230077.1	FJ807272.1
*A. cavernicola* MDC 2508	HQ436040.1	HQ442702.1
*Salmonella enterica* subsp. *enterica* ATCC 13311	NR_119108.1	EU014643.1

Available in the GenBank/NCBI database: https://www.ncbi.nlm.nih.gov//

Genetic distances and clustering were determined using Kimura’s 2-parameter model [12] and phylogenetic trees were generated by the neighbor-joining method [13] with MEGA program, version 6 [14]. Neighbor-joining tree support was evaluated with 1000 bootstrap replicates.

### Population genetic analyses

To assess the relationships among the 119 *Aeromonas* isolates and 26 reference strains, a haplotype network was constructed based on the 16S rRNA gene sequences (1454 bp) using the eBURST program version 3.0 [15, 16]. The columns containing polymorphic sites within the 16S rRNA sequence alignment were extracted, and a number N of bp was obtained for the 145 sequences analyzed.

A matrix was generated, with the numbers 1–4 replacing the adenine (A)*,* cytosine (C)*,* thymine (T) and guanine (G) bases, respectively, within the alignment and used as input. The eBURST analysis was performed using the default parameters. The network was defined as a group of closely related haplotypes, each one sharing a number N-1 of identical bases with at least one other member of this network. The stability of the relationships was assessed by bootstrap (1000 replicates).

### Detection of virulence genes

The 119 isolates were analyzed by PCR to determine the presence of the virulence genes *alt*, *ast* and *hlyA* as described [8]. The sequence of primers used to amplify the target genes: *alt* (~338 bp), *ast* (260 bp) and *hlyA* (597 bp) are listed in Table 1. To confirm the identity of the amplified segments, PCR products of three isolates from the outbreak and the reference strain *A. hydrophila* subsp. *hydrophila* ATCC 7966 were sequenced using the same PCR primers. Sequences were compared to those available at the GenBank database using the standard nucleotide-nucleotide Basic Local Alignment Search Tool (BLAST) available at https://blast.ncbi.nlm.nih.gov/Blast.cgi.

To shed some light on the virulence panorama of the *Aeromonas* isolates, we analyzed the frequency pattern for seven virulence genes: *alt*, *ast* and *hlyA* determined in the present study and *aerA*, *exu*, *lip* and *flaA*/*B*, as previously determined [8].

## Results

### Identification of the *Aeromonas* isolates at the species level

**Fig. 1:**
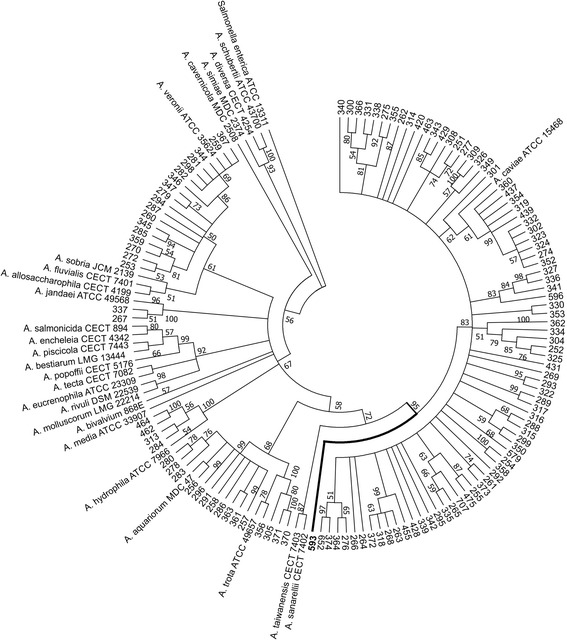
Circular phylogenetic tree (neighbor-joining) derived from concatenated 16S rRNA and *gyrB* (2374 bp) genes showing the relationship among 119 *Aeromonas* isolates, 26 known species of this genus and a *Salmonella* strain representing the outgroup. Numbers near the nodes indicate the bootstrap values ≥50 (percentage of 1000 replicates). The isolate 593 branch is highlighted

Based on the phylogenetic trees generated separately by the 16S rRNA and *gyrB* gene sequences, a total of 31% and 11% of isolates, respectively, displayed ambiguous classification (data not shown). Therefore, the phylogenetic analysis was performed applying 16S rRNA and *gyrB* (2374 bp) concatenated sequences for 146 isolates. Of these isolates, 26 were reference strains and one was an outgroup. The neighbor-joining tree is shown in Fig. 1.

In total, 118 isolates clustered with the reference strains of six *Aeromonas* species: *A. caviae* (79 isolates = 66.9%)*, A. veronii* (18 isolates = 15.3%), *A. aquariorum* (11 isolates = 9.3%), *A. hydrophila* (4 isolates = 3.4%), *A. trota* (4 isolates = 3.4%) and *A. jandaei* (2 isolates = 1.7%). All strains clustered with a reference strain in the tree with bootstrap values ≥50% (percentage of 1000 replicates). Just one environmental isolate, number 593, formed an independent branch distinct from the assessed species.

The clinical isolates fit into six species: *A. caviae* (66/103; 64.1%), *A. veronii* (18/103; 17.5%), *A. aquariorum* (11/103; 10.7%), *A. trota* (4/103; 3.9%), *A. hydrophila* (2/103; 1.9%) and *A. jandaei* (2/103; 1.9%). The environmental isolates fit into only two species: *A. caviae* (13/16; 81.3%) and *A. hydrophila* (2/16; 12.5%), plus one non-identified isolate.

### Population genetic analysis

**Fig. 2:**
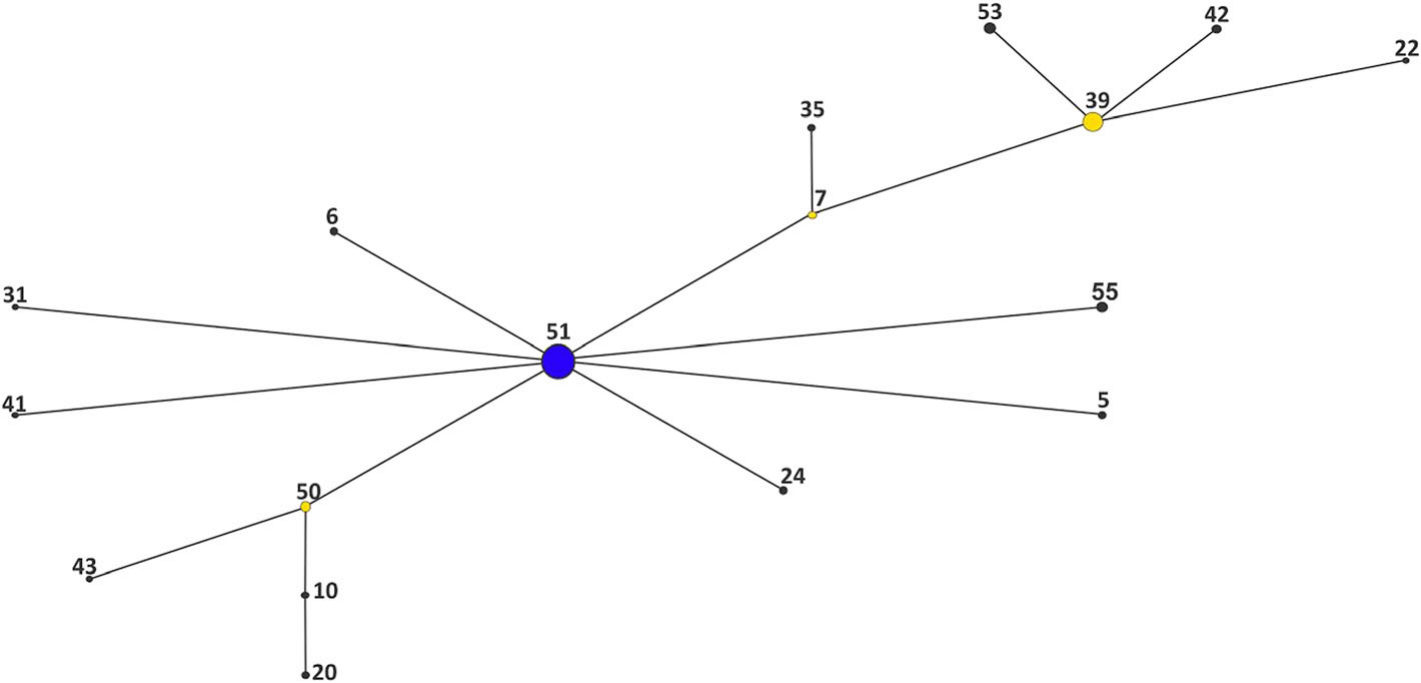
eBURST diagram based on 16S rRNA sequences from *Aeromonas* isolates and reference strains. Blue circles represent the population founder haplotype, yellow circles represent subgroup founder haplotypes, and black circles represent descending haplotypes

The relationship among the isolates was also assessed through the construction of a haplotype network based on the 16S rRNA gene sequences (1454 bp) of the 119 *Aeromonas* isolates and 26 reference strains using eBURST. This analysis clustered the 145 *Aeromonas* spp. sequences into 59 haplotypes or groups (G1-G59) as shown in Table 3. Out of these 59 haplotypes, 17 haplotypes distributed among 84 isolates and 3 reference strains clustered into a network represented by a radial diagram, in which each edge represents a nucleotide substitution (Fig. 2). 42 haplotypes distributed among 35 isolates (seven *A. caviae*, three *A. trota*, and all *A. veronii, A. hydrophila* and *A. jandaei* isolates) and 23 reference strains did not fit within the network and were categorized as satellite haplotypes.

**Table 3:** Distribution of 59 haplotypes among 119 isolates and 26 type strains of *Aeromonas* species by eBURST

Genotype	Isolates and type strains of *Aeromonas* spp*.*
1	260, 282, *A. veronii* biov. *Veronii* ATCC 35624
2	*A. schubertii* ATCC 43700
3	279
4	*A. salmonicida* subsp. *salmonicida* CECT 894
5	299, 350
6	277
7	255, 475
8	*A. sobria* JCM 2139
9	284, 313, *A. hydrophila* subsp. *hydrophila* ATCC 7966
10	278
11	*A. bestiarum* LMG 13444, *A. piscicola* CECT 7443
12	253
13	*A. rivuli* DSM 22539
14	335
15	*A. fluvialis* CECT 7401
16	*A. simiae* MDC 2374
17	*A. bivalvium* 868E
18	349
19	305
20	280
21	289
22	340
23	316
24	431
25	370, 371
26	294
27	267, *A. jandaei* ATCC 49568
28	455
29	*A. eucrenophila* ATCC 23309
30	270, 272, 287
31	579
32	346
33	317
34	341
35	373
36	359
37	*A. allosaccharophila* CECT 4199
38	*A. molluscorum* LMG 22214
39	262, 264, 266, 276, 292, 293, 308, 314, 327, 331, 334, 336, 338, 339, 364
40	285
41	322
42	356, *A. trota* ATCC 49657
43	291
44	*A. media* ATCC 33907
45	462, 464
46	259, 281, 298, 344, 345, 347, 367
47	337
48	*A. encheleia* CECT 4342
49	*A. diversa* CECT 4254
50	257, 286, 296
51	251, 254, 261, 263,265, 268, 269, 274, 275, 288, 295, 300, 301, 302, 304, 309, 315, 318, 319, 323, 324, 326, 330, 332,342, 352, 353, 354, 355, 358, 360, 362, 366, 372, 374, 420, 428, 429, 437, 439, 463, 596, 652,707, *A. caviae* ATCC 15468
52	*A. tecta* CECT 7082
53	256, 258, 283, 361, 363, *A. aquariorum* MDC 47
54	*A. popoffii* CECT 5176
55	252,325,343
56	*A. sanarellii* CECT 7402
57	593
58	*A. taiwanensis* CECT 7403
59	*A. cavernicola* MDC 2508

G51 (represented as a blue node in Fig. 2) clustered a higher number of strains (44/119; 37% of *A. caviae* isolates and the *A. caviae* ATCC 15468^T^) and is directly linked to eight derived haplotypes represented in yellow nodes in Fig. 2. G39 is the second most frequent haplotype and harbored two derived haplotypes closely related to *A. caviae*. G35 and G22 related to G7 and G39 respectively and are composed of *A. caviae* isolates. G50 and its related haplotypes (G10, G20 and G43) grouped only *A. aquariorum* isolates*.*

Some clinical and environmental *A. caviae* isolates were both closely related in the phylogenetic tree based on 16S rRNA and *gyrB* concatenated sequences and clustered into the same haplotypes in the network: 319, 354, 360, 437, 439 (G51); 374, 652 (G51); 255, 475 (G7); 265, 707 (G51).

### Detection of virulence genes

The gene *alt* was amplified in 81% (82 clinical and 15 environmental) of isolates (97/119), the gene *ast* was amplified in 11% (8 clinical and 5 environmental) of isolates (13/119), and the gene *hlyA* was amplified in 12% (11 clinical and 3 environmental) of isolates (14/119).

The identity of the three gene segments *(alt, ast* and *hlyA*) amplified from representative isolates and one reference strain was confirmed through sequencing. The alignment with sequences deposited in the GenBank database resulted in 98 to 100% identity with the corresponding genes of *Aeromonas*.

The analysis of the gene profiles based on the distribution of the seven virulence-associated genes (*aerA*, *exu, lip, flaA/B, alt, ast and hlyA*) among the clinical and environmental *Aeromonas* species revealed 29 virulence patterns and is summarized in Table 4. All the isolates were positive for at least one of the genes, and three isolates (one environmental and one clinical *A. hydrophila* and one environmental *A. caviae*) amplified all seven genes. The pattern *exu + lip + flaA/B+ alt +* was the most frequent and particular of *A. caviae* occurring among 28 isolates. The second most frequent pattern (*aer*A+ *exu + lip + flaA/B+ alt+*) was found among 13 *A. caviae,* six *A. veronii* and two *A. aquariorum* isolates (Table 4).

**Table 4:** Distribution of virulence-associated genes among 119 clinical and environmental isolates of *Aeromonas* species

	Species	*aerA*	*exu*	*lip*	*fla A/B*	*alt*	*ast*	*hly*A	No. of isolates
Clinical	*A. caviae* (*n* = 66)	−	−	−	−	+	−	−	1
		−	−	+	−	−	−	−	1
		−	+	−	−	+	−	−	2
		−	−	+	−	+	−	−	2
		−	+	−	+	−	−	−	1
		−	−	+	+	+	−	−	3
		−	+	+	−	+	−	−	7
		−	+	+	+	−	−	−	3
		−	+	−	+	+	−	−	3
		−	+	+	+	+	−	−	21
		−	+	+	+	−	+	−	3
		−	−	+	+	+	+	−	1
		+	−	+	+	+	−	−	3
		+	+	+	−	+	−	−	1
		+	+	+	+	+	−	−	11
		+	+	+	−	+	+	−	1
		−	+	+	+	+	+	−	1
		+	+	+	+	+	+	−	1
	*A. hydrophila* (*n* = 2)	+	+	−	+	+	−	+	1
		+	+	+	+	+	+	+	1
	*A. aquariorum* (*n* = 11)	−	+	−	+	+	−	+	1
		−	+	−	+	−	+	+	1
		+	−	+	+	+	−	+	2
		+	+	+	+	+	−	−	2
		+	+	+	+	+	−	+	4
		−	+	+	+	+	+	+	1
	*A. trota* (*n* = 4)	−	+	−	+	+	−	−	1
		+	+	−	+	−	−	−	2
		+	+	−	+	+	−	−	1
	*A. jandaei* (*n* = 2)	+	+	−	+	−	−	−	1
		+	+	+	+	−	−	−	1
	*A. veronii* (*n* = 18)	−	−	+	+	+	−	−	2
		+	+	+	+	−	−	−	9
		+	+	+	−	+	−	−	1
		+	+	+	+	+	−	−	6
Environmental	*A. caviae* (*n* = 13)	−	+	+	+	+	−	−	7
		−	+	+	−	+	+	−	1
		+	+	+	+	+	−	−	2
		−	+	+	+	+	+	−	2
		+	+	+	+	+	+	+	1
	*A. hydrophila* (*n* = 2)	−	+	+	+	+	+	+	1
		+	+	+	+	+	+	+	1
		+	+	+	−	+	−	−	1

## Discussion

The taxonomy, virulence potential and pathogenic mechanisms of the genus *Aeromonas* are still unclear. Therefore, in the present study, we attempted to accurately identify a set of *Aeromonas* isolates from a diarrheal outbreak in a county in Northeast of Brazil at the species level and to assess their virulence potential.

In previous work [7], all *Aeromonas* spp. isolates had been submitted to unreliable biochemical identification. Some isolates, but not all (*n* = 77), had also been classified through Restriction Fragment Length Polymorphism (RFLP) [8], but many showed atypical banding patterns. Considering this, and since the RFLP results were different from the biochemical classification for some isolates (*n* = 23), in the present work we proposed to identify the *Aeromonas* spp. isolates at the species level through a phylogenetic analysis performed on concatenated 16S rRNA and *gyrB* gene sequences.

First, we conducted the analysis with the two genes sequences individually which provided ambiguous classification of the isolates. Although 16S gene sequences can be routinely used to distinguish and establish relationships between genera and well-resolved bacterial species, this gene alone is not sufficient to classify bacteria at the species level once it did not differentiate very recently diverged species [17].

On the other hand, a phylogenetic analysis based on concatenated sequences of the 16S rRNA and *gyrB* genes identified distinct clusters of species with high bootstrap confidence values*. A. caviae* was the most prevalent species, followed by *A. veronii* and *A. aquariorum* among the clinical isolates. Among the environmental samples *A. caviae* and *A. hydrophila* were most frequently identified.

The species *A. hydrophila, A. veronii,* and *A. caviae* had been formerly categorized as major human pathogens of the *Aeromonas* genus. More recently, *A. aquariorum* has been associated with gastroenteritis [18]. Despite the pathogenic importance of *A. hydrophila*, only two clinical isolates of this species were identified in our study. On the other hand, *A. trota* and *A. jandaei* rarely reported from diarrhea isolates occurred among the study clinical isolates.

The haplotype network built by the eBURST algorithm revealed a prevalent and probably well-adapted *A. caviae* clone (G51) connected to several less prevalent variant haplotypes. Specific point mutations allowed distinguishing the *Aeromonas* species based on 16S rRNA sequences. Actually, except for the grouping of *A. bestiarum* ATCC 13444^T^ and *A. piscicola* CECT 7443^T^, no haplotype included different species, even the satellite genotypes.

The environmental isolate (number 593) was grouped in a phylogenetic branch different from the reference strains, and therefore, it is probably an unknown *Aeromonas* species. However, further studies are needed to confirm this assumption since at the time this study was conducted, not all currently described species had 16S rRNA and *gyr*B sequences deposited in the NCBI database. This analysis was also performed with *gyr*B sequences but was unsuccessful due to wide nucleotide variation.

Although their roles in human infections are not conclusively established, a number of putative virulence-associated genes were reported in several *Aeromonas* isolates [1–3]. In this work, we performed an analysis on 119 *Aeromonas* isolates for the presence and distribution of seven virulence-associated genes widely used to determine the pathogenicity potential of *Aeromonas* spp.*:* the genes coding for a cytotonic heat-labile (*alt)* and cytotonic heat-stable (*ast)* enterotoxins, a cytotoxic enterotoxin (*hly*A), the cytolysin aerolysin (*aer*A), a DNAse (*exu),* a lipase (*lip)* and a flagellin (*flaA*/*B*) were assessed.

The patterns generated by the combined presence of the *alt, ast* and *hly*A genes determined in this study and the genes *aer*A, *exu, lip* and *flaA*/*B* reported by Mendes-Marques et al. [8] produced 29 profiles according to the presence of the seven genes assessed. Taking into account the number of virulence genes present in the majority of the isolates, the species *A. hydrophila* and *A. aquariorum* exhibit higher virulence potential among the species studied, followed by *A. caviae* and *A. veronii* species. In spite of its higher prevalence among the clinical isolates compared to other species from the outbreak, *A. caviae* harbored a lower content of virulence-associated genes. However, different species could use different pathogenicity mechanisms, which may justify different gene contents. On the other hand, although some strains may harbor a certain gene, it may only be expressed in certain growth conditions [3].

## Conclusion

Taking into account their potential as a public health threat, an accurate characterization of *Aeromonas* species is crucially important. The diversity of haplotypes and their close relationship in the phylogenetic network, even between clinical and environmental isolates, suggest that *Aeromonas* spp. are well established in the study region. Furthermore, the virulence gene content highlights the potential role of these bacteria in some diarrheic processes. These results emphasize the importance of monitoring *Aeromonas* spp. in the environment to effectively treat and control any future public health event.

## Abbreviations

BHI: Brain Heart Infusion
BLAST: Basic Local Alignment Search Tool
NCBI: National Center for Biotechnology Information
PCR: Polymerase Chain Reaction
RFLP: Restriction Fragment Length Polymorphism
